# A Systematic Review of the Use of Portable Ultra-Low-Field Magnetic Resonance Imaging in Non-Acute Brain Imaging and Its Potential Use in Dementia Assessment

**DOI:** 10.1192/bjo.2024.138

**Published:** 2024-08-01

**Authors:** James Dobrzanski, Amelia Townsend, Sukhi Shergill, Joanne Rodda

**Affiliations:** 1 Kent and Medway Medical School, Canterbury, United Kingdom; 2 Kent and Medway NHS and Social Care Partnership Trust, Canterbury, United Kingdom; 3 Kings College London, London, United Kingdom

## Abstract

**Aims:**

The aims of this study were to evaluate the literature regarding the use of ultra-low-field magnetic resonance imaging (ULF-MRI) and its potential use in dementia diagnosis.

Access to neuroimaging causes bottlenecks in dementia diagnostic pathways and limits overall capacity; there is wide variation across the UK. At present, dementia diagnosis rates in the UK remain below 65% and significant improvements in efficiency and accessibility of assessment services are needed to meet growing demand.

Modern MRI scanners use high strength magnetic fields (typically 1.5–3T), are expensive to install and operate, and usually require patients to travel to a general hospital. ULF-MRI systems (typically < 0.1T) are portable, relatively inexpensive, and do not require specialist staff to operate. They do not require shielding and are powered via a standard electrical socket. The use of ULF-MRI has historically been limited by multiple factors including poor image quality. Advances in software and hardware now mean that there is realistic potential to use ULF-MRI across a range of clinical applications.

**Methods:**

The study followed the PRISMA 2020 guidelines and was registered on PROSPERO. Five electronic databases were searched for studies related to ULF-MRI using pre-developed terms. Studies comparing high field and ULF-MRI neuroimaging in adults were included. Studies of acute presentations (e.g. traumatic brain injury or acute cerebrovascular accident) were excluded. A data extraction template was used to synthesise study characteristics and outcomes. Two reviewers completed the selection process and data extraction independently.

**Results:**

2357 citations were identified, from which 101 studies were selected for further review based on title and abstract, of which eight met criteria for inclusion. Further studies were identified by forward and backward citation searching and through contact with active researchers in the field. The selected publications included both single and multi-centre studies. The Hyperfine Swoop system was used in almost all studies. Mean participant age range was 31 to 63. Qualitative and quantitative comparisons demonstrated good correspondence between high field and ULF-MRI across a range of measures studied, including volumetric measures and moderate to severe white matter hyperintensities.

**Conclusion:**

The limited available evidence suggests that there is potential for ULF-MRI to transform the approach to neuroimaging in the assessment of dementia. Dedicated research into the use of ULF-MRI in this specific application will determine if it will be one of the much-needed disruptors to our current processes of dementia assessment.

